# Medial Ganglionic Eminence Progenitors Transplanted into Hippocampus Integrate in a Functional and Subtype-Appropriate Manner

**DOI:** 10.1523/ENEURO.0359-16.2017

**Published:** 2017-04-12

**Authors:** Jui-Yi Hsieh, Scott C. Baraban

**Affiliations:** Epilepsy Research Laboratory, Department of Neurological Surgery, and Weill Institute of Neurosciences, University of California, San Francisco, CA 94143

**Keywords:** interneuron, media ganglionic eminence, optogenetics, transplantation

## Abstract

Medial ganglionic eminence (MGE) transplantation rescues disease phenotypes in various preclinical models with interneuron deficiency or dysfunction, including epilepsy. While underlying mechanism(s) remains unclear to date, a simple explanation is that appropriate synaptic integration of MGE-derived interneurons elevates GABA-mediated inhibition and modifies the firing activity of excitatory neurons in the host brain. However, given the complexity of interneurons and potential for transplant-derived interneurons to integrate or alter the host network in unexpected ways, it remains unexplored whether synaptic connections formed by transplant-derived interneurons safely mirror those associated with endogenous interneurons. Here, we combined optogenetics, interneuron-specific Cre driver mouse lines, and electrophysiology to study synaptic integration of MGE progenitors. We demonstrated that MGE-derived interneurons, when transplanted into the hippocampus of neonatal mice, migrate in the host brain, differentiate to mature inhibitory interneurons, and form appropriate synaptic connections with native pyramidal neurons. Endogenous and transplant-derived MGE progenitors preferentially formed inhibitory synaptic connections onto pyramidal neurons but not endogenous interneurons. These findings demonstrate that transplanted MGE progenitors functionally integrate into the postnatal hippocampal network.

## Significance Statement

We found that transplanted medial ganglionic eminence (MGE)-derived interneurons functionally innervate host neurons in a manner similar to interneurons derived from endogenous MGE. Developmental lineage, innervation preference, and synaptic kinetics are similar. We also found that the transplanted interneurons generate a passive inhibition on the firing activity of native pyramidal cells but not local interneurons. This inhibition only shifts but does not narrow the dynamic range of spiking, suggesting the protective effect does not interfere with normal circuit function. Together, our findings suggest that transplanted MGE-derived interneurons interact with and function in host circuits in ways mirroring native interneurons.

## Introduction

Interneuron-based cell therapy holds the potential to modify neural networks and ameliorate disease states. In recent studies, transplantation of interneuron progenitors from mouse embryonic medial ganglionic eminence (MGE) has been shown to be beneficial in various preclinical disease models, including epilepsy, associated with interneuron deficiency or dysfunction (Baraban et al., 2009; Hunt et al., 2013; Perez and Lodge, 2013; Tong et al., 2014; Tyson and Anderson, 2014). The premise underlying the success of these transplantations is that embryonic MGE progenitors are programmed to become GABAergic interneurons and specifically, parvalbumin (PV)^+^ and somatostatin (SST)^+^ interneurons (Anderson et al., 1997; Wichterle et al., 2001). Because PV^+^ and SST^+^ interneurons are strategically positioned to control network excitability, the beneficial effects of MGE transplantation are attributed to the functional synaptic integration of these transplant-derived inhibitory interneurons in the host brain. In support of this hypothesis (1) current-clamp recordings from transplant-derived cells confirm their integration as interneurons with mature firing properties, (2) voltage-clamp recordings from host pyramidal neurons in brain regions containing transplant-derived interneurons indicate a significant increase in inhibitory postsynaptic current (IPSC), and (3) electron microscopy in tissue from transplanted brains shows the formation of new synaptic connections between transplant-derived and host neurons (Alvarez-Dolado et al., 2006; Baraban et al., 2009; Calcagnotto et al., 2010; Howard et al., 2014; Southwell et al., 2014; Hunt and Baraban, 2015). While these studies are consistent with the idea that MGE progenitors are destined to integrate as inhibitory interneurons, they do not directly evaluate functional synaptic connectivity between transplant-derived interneurons and native neurons in the host brain.

To evaluate synaptic integration, it is necessary to directly study connections made between interneurons and principal cells. As mentioned, MGE progenitor cells mostly differentiate into fast-spiking PV^+^ and regular-spiking nonpyramidal SST^+^ interneurons (Wichterle et al., 2001; Xu et al., 2004; Butt et al., 2005; Du et al., 2008; reviewed by Kepecs and Fishell, 2014). We and other researchers have reported that the majority of transplanted MGE progenitor cells also derives into PV^+^ and SST^+^ interneurons regardless of the brain regions they are grafted into and that they receive excitatory input and show intrinsic properties similar to native interneurons of the same subtype (Alvarez-Dolado et al., 2006; Hunt et al., 2013; Hammad et al., 2015; Hunt and Baraban, 2015; Zhou et al., 2015; Howard and Baraban, 2016). It is widely accepted that endogenous PV^+^ interneurons primarily form fast synapses around the somatic regions of pyramidal cells, whereas endogenous SST^+^ cells mainly form slower inputs in the dendritic regions (Lee et al., 2013; Pfeffer et al., 2013). Although interneuron identities are largely controlled by the molecular programs established in the progenitor cells, whether transplant-derived PV^+^ and SST^+^ interneurons innervate postsynaptic targets and exhibit functional synaptic properties similar to their endogenous counterparts are yet to be firmly established.

**Figure 1.**
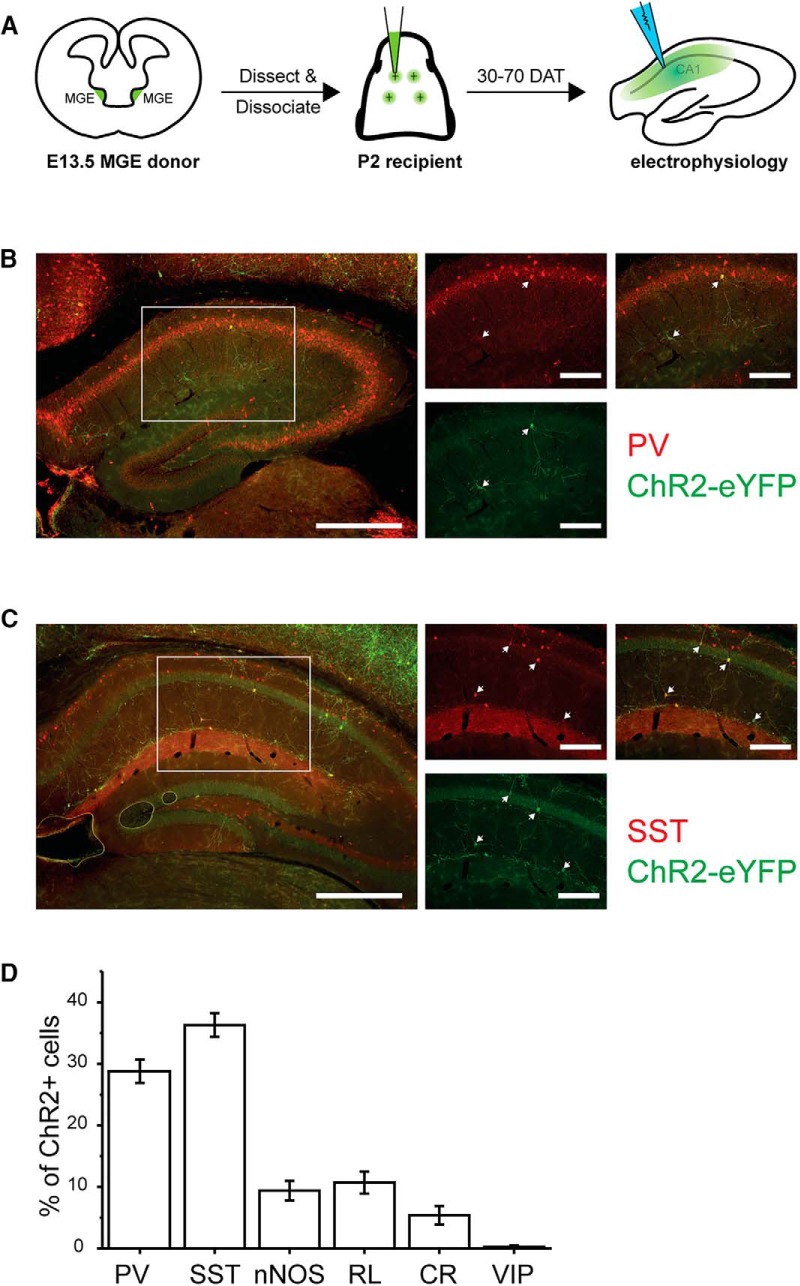
Transplanted MGE cells primarily derived into PV^+^ and SST^+^ interneurons. ***A***, Schematic illustration of the MGE transplantation. E13.5 MGE cells carrying ChR2-eYFP were harvested from donor mice and transplanted into P2 recipient pups. Immunostaining for ChR2-eYFP, PV, and SST was performed between 28 and 70 DAT, and electrophysiological recordings were conducted in the CA1 regions of hippocampi between 30 and 70 DAT. ***B***, Representative labeling for ChR2-eYFP and PV in a mouse transplanted with GAD2-ChR2 MGE aged at P2 + 43 DAT. ChR2-eYFP expression was showed in green and PV was showed in red. The image on the left shows the overview of hippocampus under 4× objective (scale bar, 500 μm). Expanded views for the boxed area were imaged under 40× objective (scale bar, 200 μm). Single-channel images for green and red are shown in the middle, and merged image is showed on the right. White arrows mark cells double-labeled. ***C***, Representative labeling for ChR2-eYFP and SST from the same animal as in ***B***. The overview of hippocampus is shown on the left (4× objective; scale bar, 500 μm). Expended views for the boxed area are shown in the middle and on the right (40× objective; scale bar, 200 μm). White arrows mark cells double-labeled. ***D***, Quantitative analysis showing the percentage of interneuron subtypes in transplanted cells. PV^+^ cells comprise 28.8 ± 1.9% (*n* = 6) of the transplanted MGE cells, whereas SST+ cells account for 36.3 ± 1.9% (*n* = 6). We also quantified the ratios for nNOS-positive (9.4 ± 1.6%), reelin-positive (10.7 ± 1.8%), CR-positive (calretinin, 5.4 ± 1.5%), and VIP-positive (0.25 ± 0.25%) cells (*n* = 4-5 animals).

To address these two issues, we investigated postsynaptic targets of MGE-derived progenitors transplanted into hippocampal networks. This was possible because of recent advances in optogenetics and establishment of interneuron-specific Cre mice, including GAD2-IRES-Cre, PV-Cre, and SST-IRES-Cre lines. These tools provide a powerful method for conditional expression of channelrhodopsin 2 (ChR2) in interneuron subpopulations and for selective stimulation of those subpopulations. MGE progenitor cells were harvested from donor mice expressing ChR2 and transplanted into the hippocampus of recipient wild-type pups. These ChR2-carrying MGE progenitor cells, depending on their genotype, are referred to here as GAD2-ChR2 MGE, PV-ChR2 MGE, and SST-ChR2 MGE, respectively. A schematic illustration of the experimental protocol is shown in Figure 1*A*. We employed whole-cell patch-clamp electrophysiology and optogenetic manipulations to identify synaptic targets of transplanted interneurons, and analyzed synaptic properties. We found that transplanted MGE-derived interneurons not only show similar selectivity in innervation as native interneurons but also generate postsynaptic currents with characteristics corresponding to their subtypes.

## Materials and Methods

### Animals and tissue transplantation

All procedures and protocols were approved by the Institutional Animal Care and Use Committee at University of California, San Francisco (protocol number AN151703). Mice were maintained under standard conditions with 12/12 h light/dark cycle, and both male and female mice were used in this study indiscriminately.

MGE transplantation was performed as previously described (Cobos et al., 2005; Alvarez-Dolado et al., 2006; Baraban et al., 2009). Briefly, MGE progenitor cells were harvested from donor embryos (embryonic day E12.2-14.5) and mechanically dissociated by pipetting in Leibovitz L-15 medium (Cell Culture Facility , University of California, San Francisco) containing 1% DNase (QIAGEN). Cells were concentrated by centrifugation and front loaded into beveled glass needles with openings between 60 and 80 μm. Stereotaxic injections into dorsal hippocampi were performed bilaterally in neonatal pups (postnatal d 1-4) anesthetized with ice (Fig. 1*A*) to minimize the number of animals required to complete these studies. Wild-type CD1 (Charles River) mice were used as recipients to transplantation. A ChR2-eYFP line (The Jackson Laboratory, #012569, also known as Ai32) was crossed with various Cre lines to generate ChR2-positive embryos, including Gad2-IRES-Cre (The Jackson Laboratory, #010802), Parv-Cre (The Jackson Laboratory, #008069), and Sst-IRES-Cre (The Jackson Laboratory, #013044). These embryos served as MGE progenitor cell donors. It is important to note that the Cre-Lox system was employed only to control the subpopulation of cells expressing ChR2 and not the type of cells transplanted. In some experiments, offspring of these ChR2-expressing mice were used as control animals in electrophysiological experiments between postnatal day P30 and 70.

## Immunohistochemistry

Wild-type CD1 mice transplanted with MGE progenitor cells harvested from Gad2-IRES-Cre;Ai32 embryos were transcardially perfused with 4% paraformaldehyde (v/v in PBS) between 28 and 70 d after transplantation (DAT). Brains were coronally sectioned at 50 μm using a vibratome (Leica VT 1000S). Sections were labeled using the following primary antibodies: chicken anti-GPF (Aves, GFP-1020 at 1:500) for ChR2-eYPF, mouse anti-PV (Sigma, P3088 at 1:500) for PV, and goat anti-SST (Santa Cruz Biotechnology, sc-7819 at 1:200) for SST. Secondary antibodies (all from Thermo Fisher) used were anti-chicken Alexa Fluor 488, anti-mouse Alexa Fluor 594, and anti-goat Alexa Fluor 594. Labeled fluorescent cells were visualized under an upright microscope (Nikon) and images were captured by a CCD camera (Andor) and NIS-elements software (Nikon).

### Electrophysiology and optogenetic stimulation

Mice were deeply anesthetized and decapitated. Brains were dissected out and sectioned at 300 μm along the coronal plane in ice-cold high-sucrose solution: 150 mM sucrose, 50 mM NaCl, 25 mM NaHCO_3_, 10 mM dextrose, 2.5 mM KCl, 1 mM NaHPO_4_-H_2_O, 0.5 mM CaCl_2_, 7 mM MgSO_4_-7 mM H_2_O. Slices were then transferred to regular artificial CSF (aCSF): 124 mM NaCl, 3 mM KCl, 1.25 mM NaHPO_4_-H_2_O, 26 mM NaHCO_3_, 10 mM dextrose, 2 mM CaCl_2_, 2 mM MgSO_4_-7 mM H_2_O, and incubated at 35**°**C for 30 min. A 45-min recovery time at room temperature was allowed before electrophysiological recording were commenced. Kynurenic acid (3 mM) was added to the aCSF in all experiments to block postsynaptic glutamate receptors.

ChR2-eYFP positive MGE-derived cells were identified in acute brain slices under an upright fluorescence microscope (Olympus). Whole-cell patch-clamp was performed in the CA1 region of hippocampi using 3-5 MΩ borosilicate electrodes on native pyramidal cells, native interneurons, or transplanted interneurons. For voltage-clamp experiments, electrodes were filled with high-chloride based internal solution: 140 mM CsCl, 1 mM MgCl_2_, 10 mM HEPES, 11 mM EGTA, 2 mM Mg-ATP, and 0.5 mM Na-GTP. Cells were held at either -50 or -60 mV in all voltage-clamp experiments. For current-clamp experiments, electrodes were filled with potassium-gluconate based internal solution: 140 mM K-gluconate, 10 mM HEPES, 1 mM NaCl, 1 mM MgCl_2_, 1 mM CaCl_2_, 5 mM EGTA, 2 mM Mg-ATP, and 0.2 mM Na-GTP. To eliminate possible complications caused by variations in resting membrane potential, cells were held at -65 mV by injecting a small amount of current. This holding current is defined as “baseline,” and all current injections in current-clamp experiments are measured relative to this baseline. All recordings were acquired using an Axon 200B amplifier, Digidata 1550A digitizer and Clampex 10.5 software (all from Molecular Device). Traces were sampled at 20 kHz and filtered at 5 kHz. Series resistance was monitored but not compensated, and cells with series resistance >20 MΩ were discarded.

A mercury lamp was used as the light source for optogenetic stimulation. A GFP and a RFP filter cube were used to isolate blue and green light, respectively. The diameter of illumination was ∼2 mm under a 40× water-immersion objective lens, and the intensity was 1–2.3 mW/mm^2^ measured by a power meter (ThorLabs). Optogenetic stimulation was time locked with electrophysiological recordings through the Digidata 1500A digitizer. The light pulse was set at 10 ms and controlled by an in-line shutter system (Uniblitz) in all experiments.

### Data analysis

Fluorescence images from immunostaining experiments were analyzed with NIS-elements software (Nikon), and colocalization of green and red fluorescent signal was identified in the software. Numbers of transplanted cells and colabeled cells were counted manually. Electrophysiological data were analyzed with Clampfit 10.5 software (Molecular Device) and Microsoft Excel (Microsoft). Data are provided as mean ± SEM. Statistical significance was assessed in OriginPro 2016 software (OriginLab). All figures were prepared in OriginPro 2016 and Adobe Illustrator (Adobe).

## Results

### Transplanted MGE-derived cells integrate as PV^+^ and SST^+^ interneurons

In rodents, transplanted embryonic MGE progenitors maintain a unique ability to migrate widely in the host brain where they differentiate into PV^+^ and SST^+^ interneurons (Wichterle et al., 1999; Butt et al., 2005; Alvarez-Dolado et al., 2006; Fogarty et al., 2007; Du et al., 2008; Hunt et al., 2013; Howard et al., 2014; Kepecs and Fishell, 2014; Hunt and Baraban, 2015). To confirm the developmental lineage of our anatomically isolated MGE progenitor cells, brains transplanted with GAD2-ChR2 MGE were stained for ChR2-eYFP, PV, and SST at 28-70 DAT. Many of the transplanted ChR2-eYFP^+^ cells colabeled for PV (Fig. 1*B*) or SST (Fig. 1*C*). Quantitative analysis showed that 28.8 ± 1.9% of the MGE-derived ChR2-eYFP^+^ cells were also PV^+^, and 36.3 ± 1.9% were SST^+^ (Fig. 1*D*). These results using ChR2 mouse donor embryos are consistent with previous studies (Alvarez-Dolado et al., 2006; Baraban et al., 2009; Southwell et al., 2012; Hunt et al., 2013; Howard et al., 2014; Sebe et al., 2014a, 2014b; Hunt and Baraban, 2015; Howard and Baraban, 2016)

### MGE-derived interneurons increase inhibition by forming functional inhibitory synapses with native neurons

**Figure 2.**
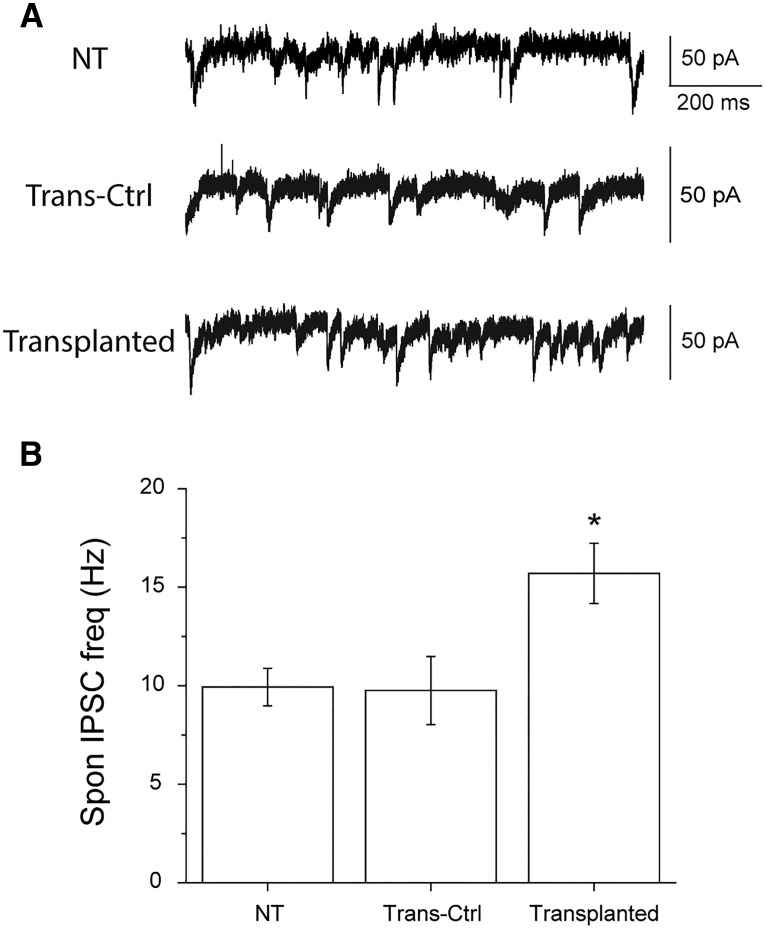
Transplanted MGE-derived interneurons increase spontaneous IPSC frequency in the hippocampus. ***A***, Representative electrophysiological recordings showing spontaneous IPSC activities. Recordings were all done in the stratum pyramidale of CA1. The frequency of IPSC recorded from a nontransplanted mouse, NT, is 14.8 Hz (top trace), whereas that from a transplanted mouse is 22.5 Hz (bottom trace). The middle trace was recorded from a pyramidal cell in transplanted mouse that received no inputs from exogenous interneurons. The lack of connection was verified by optogentically activating all nearby ChR2-expressing interneurons. This cell serves here as an internal control and referred to as Trans-Ctrl. The spontaneous IPSC frequency of it is 12.6 Hz. ***B***, Quantitative comparison in the spontaneous IPSC frequency. The average frequencies are 9.9 ± 0.9 Hz (*n* = 11), 9.8 ± 1.7 Hz (*n* = 12), and 15.7 ± 1.5 Hz (*n* = 21) for NT, Trans-Ctrl, and Transplanted, respectively. The frequency for Transplanted is significantly higher than those for the other two (one-way ANOVA, *F* = 5.541, *p* = 0.007 followed by Tukey *post hoc*, *p* = 0.029 and *p* = 0.019 for NT versus Transplanted and Trans-Ctrl versus Transplanted, respectively). There is no difference between NT and Trans-Ctrl (Tukey *post hoc*, *p* = 0.997).

In regions of cortex or hippocampus containing MGE-derived interneurons, IPSCs measured on host pyramidal neurons are increased by 20-40% compared with controls starting at 30 DAT (Calcagnotto et al., 2005; Alvarez-Dolado et al., 2006; Baraban et al., 2009). To confirm an enhancement of inhibition using ChR2-expressing donors, whole-cell voltage-clamp was performed on CA1 pyramidal cells in MGE transplanted and nontransplanted mice between 30-70 DAT. As expected, mice with MGE-derived interneurons in area CA1 exhibited a significantly higher frequency of spontaneous IPSCs compared with nontransplanted mice (Fig. 2*B*). As an internal control, we also recorded from pyramidal cells in transplanted mice that did not connect with exogenous ChR2-expressing cells. They showed the same level of spontaneous IPSCs as those seen in nontransplanted mice (Fig. 2*B*). This observation strongly suggests the enhancement in inhibition is a direct result of the exogenous MGE-derived interneurons rather than a nonspecific effect.

**Figure 3.**
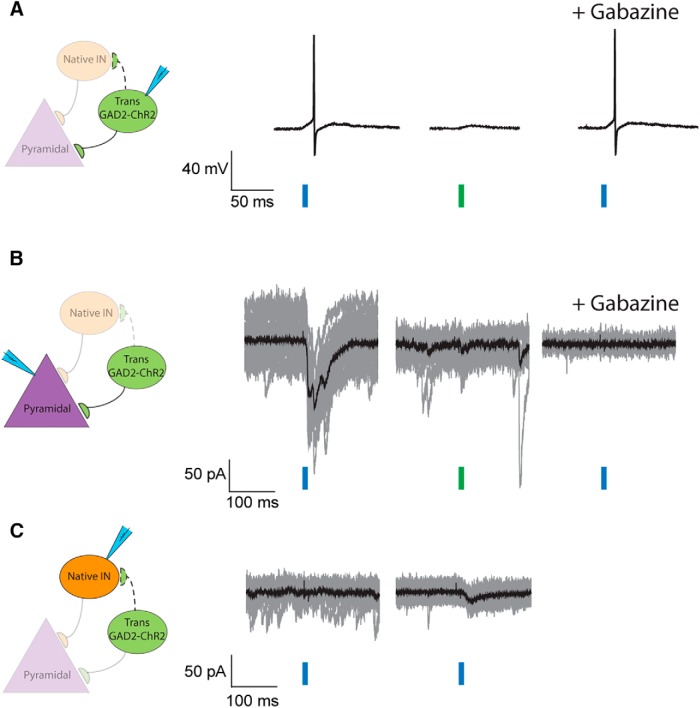
Activation of transplanted cells evokes consistent IPSC responses in native pyramidal cells but not native interneurons. ***A***, Electrophysiological traces obtained from a transplanted ChR2-expressing cell. A brief 10-ms blue-light pulse elicited an AP (left trace) that was insensitive to the subsequent addition of 100 μM gabazine (right trace). Application of green-light pulses to the same cell failed to bring this interneuron over threshold (middle trace). Recordings from three other transplanted cells show the same result (data not shown). ***B***, Traces recorded from a native pyramidal cell in the vicinity of transplanted interneurons. While brief blue-light pulses consistently evoked IPSC responses (left traces), green-light pulses failed to do the same (middle traces). Subsequent application of 100 μM gabazine to the same cell compromised blue-light pulses and blocked the generation of IPSCs (right traces). Traces for single trials are shown in gray and the averaged response of the cell is showed in black. We recorded a total of 11 cells and all showed consistent responses to blue light. ***C***, Recordings obtained from native interneurons. Blue-light pulses did not generate consistent IPSCs in 15 out of 16 native interneurons. Recordings from one of these six cells are shown on the left. Only one of the 16 interneurons recorded showed small light-dependent responses (right traces). They were drastically smaller and slower compared with blue-light responses shown in pyramidal cells (see also text in the Results).

A plausible and likely interpretation for the enhancement of GABA-mediated inhibition consistently observed with MGE transplantation (Calcagnotto et al., 2005; Alvarez-Dolado et al., 2006; Baraban et al., 2009; Fig. 2) is that MGE-derived interneurons make functional inhibitory synapses onto native pyramidal cells. To directly test this hypothesis, we used optogenetics to photostimulate MGE-derived interneurons carrying ChR2, and then monitored light-evoked responses in native pyramidal neurons or interneurons in area CA1. Brief 10 ms blue-light pulses consistently elicit action potentials (APs) >40 mV in amplitude on GAD2-ChR2-expressing interneurons (*n* = 4; Fig. 3*A*). The number of APs evoked varied slightly between cells, presumably associated with the differential excitability of interneuron subtypes and cell-to-cell variability in the level of GAD2-ChR2 expression. However, the number of APs evoked was consistent within the same cell. Light-evoked APs were not sensitive to the addition of a GABA-receptor antagonist (100 μM gabazine; Fig. 3*A*). Since we also included a nonspecific glutamate receptor blocker (3 mM kynurenic acid) in the aCSF for all recordings (see also Materials and Methods), these observations suggest that light-evoked responses are intrinsic to the ChR2-expressing neurons. Brief 10-ms green-light pulses did not generate APs (Fig. 3*A*). When recording from native pyramidal cells, evoked IPSCs ranged from 10.5 to 403.3 pA in amplitude (*n* = 11) and were consistently observed immediately after blue-light pulses, whereas green-light pulses did not elicit responses (Fig. 3*B*). All light-evoked IPSCs were eliminated by 100 μM gabazine indicating a dependence on GABA receptor-mediated synaptic inputs. Taken together, these findings suggest that transplanted MGE-derived interneurons increase the level of inhibition in hippocampal circuits by forming functional inhibitory synapses with native neurons.

**Figure 4.**
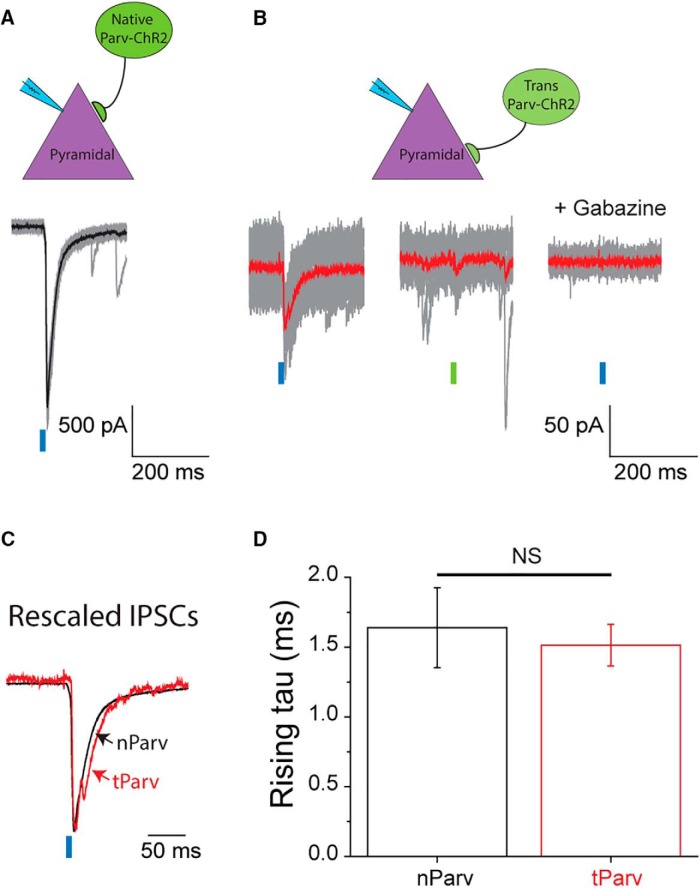
Transplanted and native PV^+^ interneurons generate IPSCs with comparable rising kinetics. ***A***, Representative recordings from a native pyramidal cell in a mouse expressing ChR2-eYFP in all the PV^+^ interneurons. Blue-light pulses evoked fast and significant IPSC responses consistently. Traces for single trials are shown in gray and the averaged response is presented in black. The amplitude of the averaged IPSC for this cell is 1683.4 pA. Recordings from nine other cells also showed consistently light-dependent IPSCs that ranged from 204.8 to 1817.1 pA. ***B***, Recordings from a native pyramidal cell in a Parv-ChR2 MGE transplanted mouse. Traces for single trials are shown in gray and the averaged response is presented in red. Blue-light pulses evoked consistent and fast IPSCs with averaged amplitude of 78.8 pA (left traces), whereas green-light pulses failed to do so (middle traces). Applying 100 μM gabazine to the same cell blocked all IPSC activities, either light dependent or independent (right traces). We recorded eleven other native pyramidal cells, and all of them showed the same result. The averaged IPSC amplitude ranged from 19.1 to 181.4 pA. ***C***, Averaged IPSC responses to blue-light from ***A*** and ***B*** were rescaled and superimposed. The black trace (IPSC from a native PV^+^ cell, nParv) shares similar rising kinetics with the red trace (IPSC from a transplanted PV^+^ cell, tParv). ***D***, The averaged rising τ for nParv and tParv are 1.64 ± 0.29 ms (*n* = 10) and 1.51 ± 0.16 ms (*n* = 12), respectively, and they are not significantly different (two-sample *t* test, *p* = 0.678).

**Figure 5.**
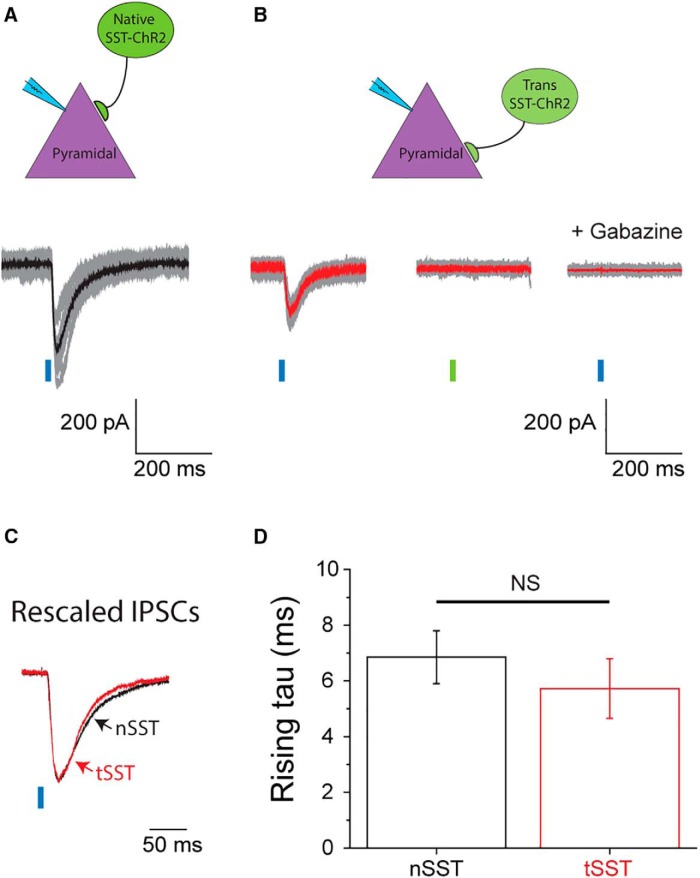
Transplanted and native SST^+^ interneurons generate IPSCs with comparable rising kinetics. ***A***, Representative recordings from a native pyramidal cell in a mouse expressing ChR2-eYFP in all the SST^+^ interneurons. Blue-light pulses evoked consistent IPSCs. Traces for single trials are in gray and the averaged response is in black, for which the amplitude is 1683.4 pA. Recordings from nine other cells also showed consistent light-dependent IPSCs (52.5-464.2 pA). ***B***, Recordings from a native pyramidal cell in a mouse transplanted with SST-ChR2 MGEs. Traces for single trials are shown in gray and the averaged response in red. Blue-light pulses evoked consistent IPSCs averaged at 78.8 pA (left traces), whereas green-light pulses failed to elicit any notable responses (middle traces). Applying 100 μM gabazine to the same cell blocked all IPSC activities (right traces). Seven other native pyramidal cells show the same responses to light and to gabazine. The averaged IPSC amplitude recorded from them ranged from 51.7 to 187.3 pA. ***C***, Averaged IPSC responses to blue-light from ***A*** and ***B*** were rescaled and superimposed. The black trace (IPSC responded to a native SST^+^ cells, nSST) the red trace (IPSC responded to a transplanted SST^+^ cell, tSST) show comparable rising kinetics. ***D***, The averaged rising τ for nSst is 6.80 ± 0.90 ms (*n* = 10) and that for tSST is 5.71 ± 1.10 ms (*n* = 8). They are not significantly different (two-sample *t* test, *p* = 0.414).

Physiologically, MGE-derived interneurons primarily innervate neurons in stratum pyramidale and stratum lacunosum moleculare, but not nearby interneurons in the stratum oriens (Elfant et al., 2008; Lovett-Barron et al., 2012; Bezaire and Soltesz, 2013; Kepecs and Fishell, 2014). GABA-mediated inhibition onto native interneurons in regions containing MGE-derived interneurons was not found to be enhanced in cortex (Baraban et al., 2009). These findings are consistent with our interpretation that MGE-derived interneurons selectively innervate native pyramidal neurons following transplantation. To directly evaluate functional inhibitory connectivity between MGE-derived interneurons and host brain neurons, we also recorded from native interneurons in stratum oriens while optogenetically activating the MGE-derived GAD2-Chr2-expressing interneuron subpopulation. Photostimulation of MGE-derived interneurons did not consistently evoke IPSCs in native interneurons (*n* = 16; Fig. 3*C*). Indeed, a light-evoked IPSC response was only observed on one native interneuron. As shown in Figure 3*C*, the amplitude of light-evoked events in this single interneuron were dramatically smaller (∼15 pA) than those in Figure 3*B* (∼80 pA) recorded from a native pyramidal cell. IPSC rising kinetics (τ = 16.3 ms) of this interneuron was much slower than those observed in pyramidal cells (Fig. 3*B*; see also Figures 4*D* and 5*D* ). Thus, these results are also consistent with our hypothesis that MGE-derived interneurons primarily make functional inhibitory connections with native pyramidal neurons.

### Transplanted and native interneurons share comparable IPSC kinetics in a cell-type-specific manner

Endogenous PV fast-spiking interneurons primarily innervate somatic regions of pyramidal neurons and exhibit fast IPSC rise time kinetics, whereas SST interneurons largely innervate dendrites and exhibit slower IPSC rising kinetics (Lee et al., 2013; Pfeffer et al., 2013). Whether exogenous MGE-derived PV^+^ and SST^+^ interneurons integrate in the host circuit in a similar manner is not known. To investigate the functional connections made by MGE-derived interneuron subpopulations, we generated MGE donors expressing ChR2-eYFP in either PV^+^ or SST^+^ cells for transplantation into recipient CD1 pups. This approach allowed us to characterize the functional integration of MGE-derived interneuron subtypes in a cell-type specific manner. For control studies, we used age-matched nontransplanted mice expressing ChR2-eYFP driven by PV- or SST-Cre promoters, referred to here as native Parv-ChR2 (nParv-ChR2) and native SST-ChR2 (nSST-ChR2) respectively; transplanted PV^+^ and SST^+^ cells carrying ChR2-eYFP are referred to as tParv-ChR2 and tSST-ChR2, respectively. Photostimulation of nParv-ChR2 interneurons consistently generated large amplitude IPSCs in native pyramidal cells (ranged from 204.8 to 1817.1 pA, *n* = 10; Fig. 4*A*). Light activation of tParv-ChR2 cells also evoked IPSC responses in native pyramidal cells but with smaller amplitudes (ranged from 19.1 to 181.4 pA, *n* = 12), consistent with the presence of fewer MGE-derived PV^+^ interneurons (compared with native interneurons) in the host brain. A representative cell is shown in Figure 4*B*. Green-light pulses did not elicit IPSCs and 100 μM gabazine eliminated both light-dependent and independent IPSCs. Despite the difference in amplitude, the rising kinetics of light-evoked IPSCs showed no differences between nParv-ChR2 and tParv-ChR2 cells. Both generated IPSCs with fast onset kinetics in pyramidal cells with a rising time constant of ∼1.5 ms (1.6 ± 0.3 for nParv-ChR2, *n* = 12; 1.5 ± 0.1 for tParv-ChR2, *n* = 10; Fig. 4*D*).

Next, we recorded postsynaptic responses of native pyramidal cells following photostimulation of nSST-ChR2 cells. A representative cell is shown in Figure 5*A*. Consistent IPSCs were observed immediately after blue-light pulses. The rising kinetics of these responses were smaller and slower than that from the nParv-ChR2 cell shown in Figure 4*A* (τ = 5.2 for black trace in Figure 5*A*; τ = 1.8 ms for black trace in Figure 4*A*). The amplitude of IPSCs from nSST-ChR2 inputs ranges from 52.3 to 464.2 pA (*n* = 10), and the average time constant of rising was 6.8 ± 0.9 ms, which is 4.3 times slower than the average from nParv-ChR2 at 1.6 ± 0.3 ms (Figs. 4*D*, 5*D* ). This finding is consistent with observations that PV^+^ interneurons generate faster postsynaptic currents in pyramidal cells than SST^+^ interneurons (Lee et al., 2013; Pfeffer et al., 2013). Activation of transplanted SST^+^ cells also generated consistent IPSCs in pyramidal cells, which could be blocked by subsequent addition of 100 μM gabazine. IPSCs elicited by tSST-ChR2 cells have amplitudes ranging from 51.7 to 187.3 pA (*n* = 8), and their averaged time constant of rising is 5.7 ± 1.1 ms, which is not significantly different from that of nSST-ChR2 (Fig. 5*D*). Taken together, our results show that transplanted cells, either PV^+^ or SST^+^, generate subtype specific postsynaptic responses in pyramidal cells, mirroring those generated by native interneurons of the same subtype.

### Transplanted interneurons introduce inhibitory gain control to native pyramidal cells without altering their spiking behaviors

**Figure 6.**
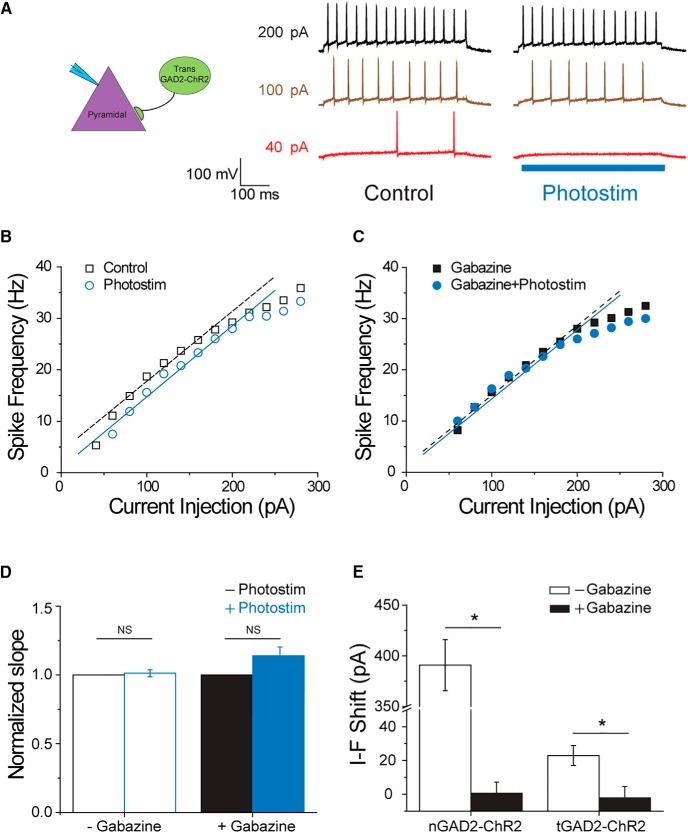
Activation of transplanted interneurons introduce inhibitory gain control to native pyramidal cells. ***A***, Representative traces recorded from a native pyramidal cell at various levels of current injection in the absence and presence of photostimulation. In the absence of photostimulation, 40-pA current injection (red trace) brought the cell over threshold and generated two APs, giving a frequency of 5 Hz; 100-pA (brown trace) and 200-pA (black trace) current injection induced higher spiking frequency at 18.7 and 29.2 Hz, respectively. With simultaneous blue-light photostimulation, 40 pA was no longer sufficient to evoke AP, and the spike frequencies induced by 100 and 200 pA were reduced to 15.6 and 28 Hz, respectively. ***B***, ***C***, I-F curves of the cell showed in ***A*** under various conditions. Curves were fit linearly with a data range picked manually. The slope factor of the fit line for control condition is 0.136 (open squares, *r* ^2^ = 0.955), whereas the slope factor with photostimulation is 0.138 (open circles, *r* ^2^ = 0.990). After the addition of 100 μM gabazine, the slope factor is 0.136 (closed squares, *r* ^2^ = 0.988), and that with photostimulation is 0.135 (closed circles, *r* ^2^ = 0.970). By comparing the fit lines, we found that photostimulation right shifts the curve by 23.7 pA, which can be reversed by the addition of gabazine. The shift in the presence of gabazine is 3.6 pA. ***D***, The slope factors from fit results were normalized, averaged, and compared. In the absence of gabazine, the normalized slope factor with photostimulation is 1.013 ± 0.026, which is not different from the control group (paired *t* test, *n* = 9, *p* = 0.630). In the presence of 100 μM gabazine, the normalized slope factor with photostimulation is 1.125 ± 0.066 and is not significantly different from that without photostimulation (paired *t* test, *n* = 5, *p* = 0.334). ***E***, Parallel shifts in I-F curves were compared with and without gabazine. On average, activation of transplanted interneurons, tGAD2, right shifts the I-F curve by 21.1 ± 5.5 pA, which can be rectified by 100 μM gabazine to -2.2 ± 6.8 pA (two-sample *t* test, *n* = 9 and 5, *p* = 0.023). The minor shift in the presence of gabazine is not different from 0 (one-sample *t* test, *p* = 0.661). Similarly, activation of native interneurons, nGAD2, shifts the I-F curves to the right (342.8 ± 30.3 pA, *n* = 5), and subsequent addition of 100 μM gabazine completely eliminated the effect (-5.4 ± 7.5 pA after gabazine, *n* = 4; two-sample *t* test, *p* = 0.00002). The shift recorded in the presence of gabazine is not different from 0 (one-sample *t* test, *p* = 0.460).

To better understand the nature of inhibition introduced by transplantation and functional impact on local circuits, we generated current-frequency (I-F) curves for native pyramidal cells in regions containing MGE-derived GAD2-ChR2-expressing interneurons. Figure 6*A* shows representative recordings from a native pyramidal cell innervated by MGE-derived interneurons. In the absence of photostimulation, a 40-pA current injection elicited two APs giving a spiking frequency of 5 Hz; with 100- and 200-pA current injections, the frequencies elicited were 18.7 and 29.2 Hz, respectively. With the presence of blue light stimulating the transplanted MGE-derived interneuron population, the same 40-pA current injection no longer generated any AP in the recorded pyramidal cell (see red traces in Figure 6*A*). The spike frequency was lowered slightly at 100-pA current injection from 18.7 to 15.6 Hz, whereas that at 200-pA injection stayed almost unaffected (29 Hz vs 28 Hz; Fig. 6*A*). Further analysis showed that photostimulation caused a right shift in I-F curve of this cell, which could be reversed by the addition of 100 μM gabazine (Fig. 6*B*,*C*). The I-F curves were fit with linear regression. The data range used for fitting was selected manually for each neuron so that *r* ^2^ > 0.95, except in a few cases, it was between 0.9 and 0.95. Slope factors and *x*-intercepts (the axis for current injection) from the fitting results were analyzed and compared between recording conditions: (1) with or without photostimulation, and (2) with or without gabazine. We found that on average photostimulating transplanted interneurons right shifts the I-F curve by 21.1 ± 5.5 pA without significantly affecting the slope factor (*n* = 9; Fig. 6*D*,*E*). The addition of gabazine completely blocked the effect, and reversed the shift to −2.2 ± 6.8 pA, which is not significantly different from no-shift (*n* = 5, one sample *t* test, *p* = 0.661).

To confirm that this parallel shift in I-F curve by exogenous interneurons is a physiologic function, we tested whether native interneurons also showed a similar phenomenon. We repeated the experiments and recorded from pyramidal cells in nontransplanted mice carrying ChR2 in all interneurons (Gad2-IRES-Cre;Ai32). This allows us to investigate the connections formed physiologically between native interneurons and pyramidal neurons. Photostimulation significantly shifted the I-F curve to the right by 342.8 ± 30.3 pA (*n* = 5; Fig. 6*E*) without affecting the slope factors (data not shown). Again, this rightward shift could be reversed by the addition of gabazine. Thus, transplanted MGE-derived interneurons appear to exert gain control of pyramidal cell firing in a manner functionally similar to native interneurons.

**Figure 7.**
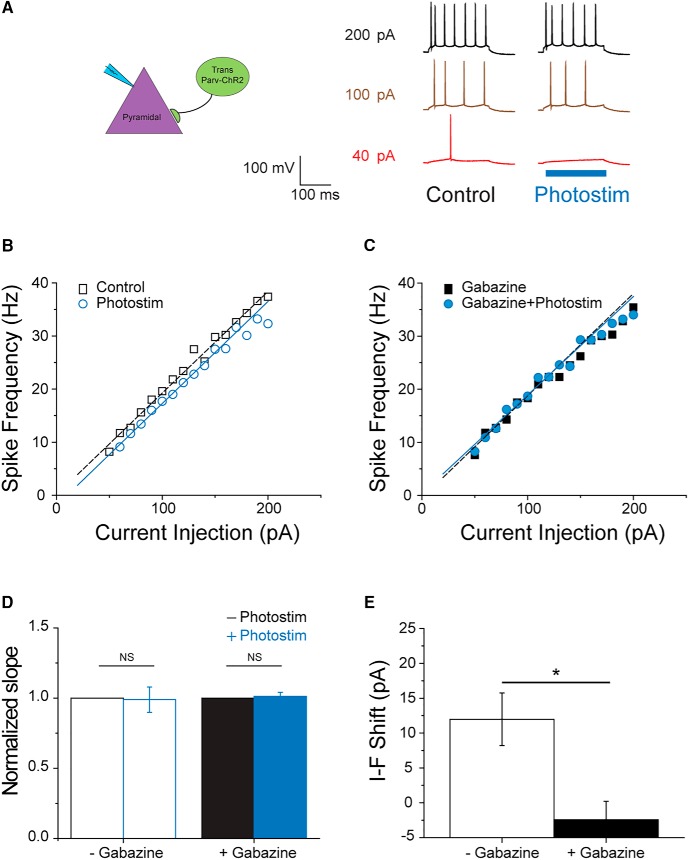
Transplanted PV^+^ interneurons contribute to the inhibitory gain control on native pyramidal cells. ***A***, Representative traces recorded from a native pyramidal cell at various levels of current injection with and without simultaneous activation of nearby transplanted PV^+^ cells. In the absence of photostimulation, 40-pA current injection elicited single AP over a 200-ms period (red trace). When current injection was increased to 100 and 200-pA, the multiple spikes were elicited, and the frequencies were 19.6 Hz (brown trace) and 37.4 Hz (black trace), respectively. In the presence of photostimulation, 40-pA current injection failed to bring the cell over threshold. On 100- and 200-pA current injection, the spike frequencies were 17.7 and 35.4, respectively. ***B***, ***C***, The I-F curves under various conditions obtained from the same cells shown in ***A***. Linear regression was employed to fit the data. The slope factor for the control condition is 0.118 (open squares, *r* ^2^ = 0.978), and that with photostimulation is 0.110 (open circles, *r* ^2^ = 0.982). After the application of 100 μM gabazine, the slope factors are 0.127 (closed squares, *r* ^2^ = 0.972) and 0.127 (closed circles, *r* ^2^ = 0.992) for conditions with and without photostimulation, respectively. In the absence of gabazine, photostimulation shifts the I-F curve to the right by 10.6 pA, whereas it only shifts the curve by 4.4 after the addition of gabazine. ***D***, In the absence of gabazine, the normalized slope factor with photostimulation is 0.989 ± 0.090 and is not significantly different from that under control condition (paired *t* test, *n* = 8, *p* = 0.907). In the presence of 100 μM gabazine, the normalized slope factor with photostimulation is 1.013 ± 0.029 compared with that without photostimulation. There is no statistical significance between these two conditions (paired *t* test, *n* = 6, *p* = 0.399). ***E***, On average, activating transplanted Parv^+^ cells shift the I-F curve of pyramidal cells by 12 ± 3.8 pA (*n* = 8). This effect can be significantly reduced by 100 μM gabazine (-2.4 ± 2.6 pA, *n* = 6; two sample *t* test, *p* = 0.013). The shift observed in the presence of gabazine is not different from zero (one-sample *t* test, *n* = 6, *p* = 0.252).

To further identify the interneuron subtype-specific source of this inhibitory shift, we performed current-clamp experiments on pyramidal cells while selectively activating either tParv-ChR2 or tSST-ChR2 cells. We found that while 40-pA current injection was sufficient to bring a pyramidal cell over AP threshold, it failed to do so with simultaneous activation of nearby tPV-ChR2 cells (Fig. 7*A*). This effect was much less obvious when the level of current injection was raised to 100 and 200 pA, with which the difference in spiking frequencies was <5% with and without blue-light activation (19.7 and 17.7 Hz at 100-pA injection; 37.4 and 35.4 Hz at 200-pA injection; Fig. 7*A*). By further analyzing the I-F curve, we found that photostimulation shifted the curve to the right, and the addition of 100 μM gabazine reversed the effect (Fig. 7*B*,*C*). We found that, on average, activating tParv-ChR2 cells right shifts the I-F curve of pyramidal cells by 12 ± 3.8 pA without significantly altering slope factors (*n* = 8) and that this parallel shift can be completely eliminated by the addition of gabazine (*n* = 6; Fig. 7*D*,*E*).

**Figure 8.**
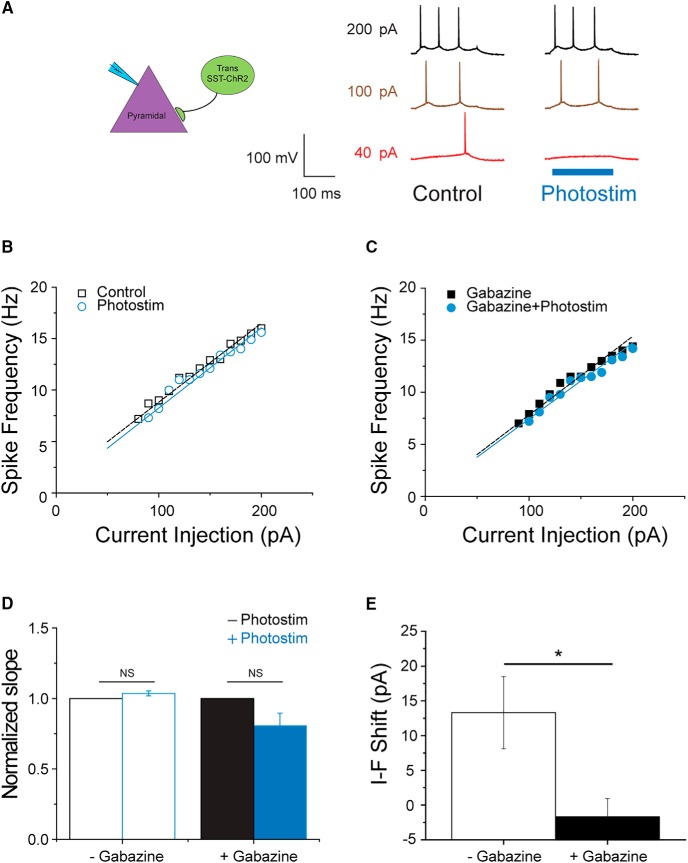
Transplanted SST^+^ interneurons also contribute to the introduced inhibition on native pyramidal cells. ***A***, Representative traces recorded from a native pyramidal cell at various levels of current injection. Without photostimulating transplanted SST^+^ cells, 40 pA of current injection elicited a single AP over 200 ms (red trace). The spike frequencies under 100-pA (brown trace) and 200-pA current injection (black trace) are 9 and 16 Hz, respectively. With simultaneous photostimulation, 40 pA was no longer sufficient to elicit APs, and the frequencies evoked by 100 and 200 pA were reduced to 8.2 and 15.6 Hz. ***B***, ***C***, The I-F curves under various conditions obtained from the same cells shown in ***A***. Data were fit with linear regression to obtain slope factors and intercepts along the *x*-axis (current axis). The slope factor for the control condition is 0.077 (open squares, *r* ^2^ = 0.969), and that with photostimulation is 0.078 (open circles, *r* ^2^ = 0.946). After 100 μM gabazine, the slope factors are 0.065 (closed circles, *r* ^2^ = 0.967) and 0.069 (closed squares, *r* ^2^ = 0.975) for conditions with and without photostimulation, respectively. In the absence of gabazine, photostimulation right shifts the I-F curve by 9.1 pA in this cell, whereas it only shifts the curve by 4.4 after the addition of 100 μM gabazine. ***D***, In the absence of gabazine, the normalized slope factor with photosimulation is 1.036 ± 0.015 and is not significantly different from that under control condition (paired *t* test, *n* = 6, *p* = 0.079). In the presence of 100 μM gabazine, the normalized slope factor with photostimulation is slightly lower (0.806 ± 0.103, *n* = 5) than that without photostimulation. However, this decrease is not statistically significant (paired *t* test, *n* = 5, *p* = 0.073). ***E***, Photostimulating transplanted SST^+^ interneurons shifts the I-F curve of pyramidal cells by 13.3 ± 5.2 pA (*n* = 6), which can be compromised by 100 μM gabazine (-1.7 ± 2.6 pA, *n* = 5; two sample *t* test, *p* = 0.037). The shift observed by photostimulation in gabazine is not different from zero (one-sample *t* test, *n* = 5, *p* = 0.513).

We next tested the functional impact of inhibition from tSST-ChR2 on pyramidal cells. Similar to the examples shown earlier in this study, 40-pA current injection elicited an AP in a pyramidal cell, and simultaneous activation of tSST-ChR2 cells compromised this spike. This suppression was nearly absent when the level of current injection was at 100 or 200 pA. The impact on spiking frequency was very subtle if any (Fig. 8*A*). The shift in I-F curve by photostimulation was 9.1 pA in the cell shown in Figure 8*A*, and 100 μM gabazine blocked the shift completely (Fig. 8*B*,*C*). The averaged right shifts by photostimulation are 13.3 ± 5.2 and 1.7 ± 2.6 pA without and with gabazine, respectively (Fig. 8*E*). The latter is not different from zero (one-sample *t* test, *n* = 5, *p* = 0.513), suggesting the expected indifference of I-F curve to light in the presence of synaptic blocker, gabazine. Photostimulation did not change the slope factor of I-F curves regardless the presence or absence of gabazine (Fig. 8*D*). Our results suggest that both transplanted PV^+^ and SST^+^ interneurons contribute to the introduction of gain control following MGE transplantation.

## Discussion

Inhibitory interneurons constitute ∼20% of the neuronal population in cortical networks. In general, interneuron synaptic interactions are short range and dense. Mature PV^+^ interneuron axons target proximal dendrites and somata of pyramidal cells, close to where APs are generated. Thus, their postsynaptic inhibitory effect is both powerful and precisely coordinated. Mature SST^+^ interneurons are often dendritic-targeting and suppress dendritic calcium spikes and bursting (Murayama et al., 2009) but have also been shown to modulate pyramidal neuron firing (Lovett-Barron et al., 2012). These properties of interneurons derived from MGE progenitors could explain the beneficial effects seen when embryonic MGE is transplanted into various disease models associated with interneuron deficiency or dysfunction (Baraban et al., 2009; Hunt et al., 2013; Perez and Lodge, 2013; Tong et al., 2014; Tyson and Anderson, 2014). Indeed, the most parsimonious interpretation of these results coincides with a concept first put forth by Prince and Wilder of an “inhibitory surround” (Prince and Wilder, 1967; Traub, 1983) near the seizure focus that constrains the spread of an epileptic discharge. Theoretical calculations show that if the GABA-mediated inhibition is sufficiently powerful, it can effectively suppress any amount of excitatory drive (Trevelyan and Watkinson, 2005). However, other mechanisms explaining MGE transplantation have been put forth: (1) reorganization of host circuitry by introducing a new set of weak inhibitory synapses (Southwell et al., 2010; Southwell et al., 2014) or (2) addition of “young” interneurons thus weakening endogenous inhibition (Davis et al., 2015). Experimental support for transplanted MGE-derived interneurons providing increased synaptic inhibition and enhanced inhibitory surround requires a direct demonstration that these progenitors differentiate into mature PV^+^ and SST^+^ interneurons making selective inhibitory synaptic connections within the host network. Using a combination of *in vitro* electrophysiology and optogenetic techniques we provide that evidence here.

### Transplanted and endogenous MGE-derived interneurons share common synaptic targets and physiology in a subtype-specific manner

For synaptic integration to be the working mechanism underlying a feasible cell-therapy, there are three indispensable requirements: (1) the developmental lineage of transplanted MGE cells is autonomous, (2) the innervation targets of exogenous interneurons are inherently the same as those of endogenous interneurons, and (3) the synaptic physiology of exogenous interneurons are comparable to that of endogenous interneurons. The first requirement is crucially important because interneuron subtypes have distinct physiology and differential innervation preferences that may provide opposite effects to local circuits. For instance, innervation on native pyramidal cells offers inhibition, whereas that on native interneurons generates dis-inhibition, which contradicts the goal of enhancing inhibitory surround. Interneuron complexity, however, raises the possibility that transplant-derived interneurons could integrate in a random fashion with inappropriate and detrimental consequences for circuit modification. Prior work suggests that differentiation of transplanted embryonic MGE progenitors is autonomous (Butt et al., 2005; Alvarez-Dolado et al., 2006; Fogarty et al., 2007; Du et al., 2008; Hunt et al., 2013; Howard et al., 2014; Kepecs and Fishell, 2014; Hunt and Baraban, 2015; Howard and Baraban, 2016). Here, we also found that transplanted MGE progenitors primarily derived into PV^+^ and SST^+^ cells (Fig. 1*B--D*), and formed functional synapses with host neurons (Fig. 2).

As a means to control or modulate network excitability, the primary innervation targets for endogenous MGE-derived interneurons are excitatory pyramidal neurons (Elfant et al., 2008; Lovett-Barron et al., 2012; Bezaire and Soltesz, 2013; Kepecs and Fishell, 2014). It is important that transplanted interneurons maintain this signature attribute and do not reorganize the host network or target nearby native interneurons. Baraban et al. (2009) demonstrated there was no elevation in spontaneous IPSC frequency onto native interneurons following transplantation into cortex. Here, we employed optogenetic tools to more carefully evaluate potential connections between transplant-derived and native interneurons. Compared with the previous study, this approach is more sensitive because the transplanted ChR2-expressing interneurons were directly activated to elicit measurable postsynaptic responses. Consistent with Baraban et al. (2009), we found that native pyramidal neurons showed robust and gabazine-sensitive IPSCs (Fig. 3) in response to activation of transplanted interneurons. Among all the native interneurons recorded, only one was found to connect with transplant-derived interneurons, and the response was very small (see also Results). The rarity of this type of connection is consistent with the interpretation that MGE-derived interneurons do not innervate other interneurons and preferentially make connections with pyramidal cells. It is also possible a very small number of transplant-derived interneurons arise from the caudal ganglionic eminence (CGE; a region generating interneuron-innervating interneurons; Nery et al., 2002; Xu et al., 2004; Butt et al., 2005; Cobos et al., 2005; Miyoshi et al., 2010; Kepecs and Fishell, 2014), as these are anatomic embryonic subdissections and could include a small piece of CGE tissue. Furthermore, the fact that transplant-derived and native interneurons share common geographic area and yet form virtually no synapses argues against the idea that exogenous interneurons reorganize the host network and provide nonspecific inhibition to their immediate surroundings. Taken together, our data strongly suggest that transplanted interneurons show an inherent innervation preference mimicking endogenous MGE-derived interneurons.

Although we and several studies have reported that MGE-derived interneurons show comparable intrinsic properties to endogenous interneurons following transplantation (Fig. 1*D*,*E*; Alvarez-Dolado et al., 2006; Hunt et al., 2013; Howard et al., 2014; Sebe et al., 2014b; Zhou et al., 2015), it is not yet settled whether they also have proper synaptic characteristics matching their interneuron subtypes. Comprehensive recapitulation of functional features is essential to any type of cell-based therapy. Either gain-of-function or loss-of-function may lead to unwanted effects. Recently, Howard and Baraban (2016) presented a detailed functional comparison between transplant-derived and native PV^+^ interneurons. By performing dual whole-cell patch-clamp from PV^+^ and pyramidal cells, they reported that in mouse cortex transplanted PV^+^ interneurons (7-35 DAT) showed delayed but identical characteristics as native PV^+^ cells during maturation, including their intrinsic properties, excitatory synaptic inputs, and postsynaptic properties. Here, however, we focused on a functional comparison after transplanted neurons are mature (30-70 DAT), and we took one step further to distinguish both PV^+^ and SST^+^ cells. We found that MGE transplant-derived interneurons formed appropriate connections with postsynaptic targets matching their native interneuron subtypes in hippocampus, e.g., PV^+^ cells generated fast IPSCs on pyramidal cells and SST^+^ cells generated slow IPSCs. More importantly, kinetic properties of these postsynaptic currents were comparable to that from native interneurons of the same subtype (Figs. 4, 5). These findings extend and confirm our previous PV^+^ interneuron study (Howard and Baraban, 2016). Taken together, we conclude that transplant-derived and endogenous MGE-derived interneurons are fundamentally the same, sharing a common developmental lineage, innervation preference, and synaptic function.

### Inhibitory gain-control generated by transplanted interneurons sheds light on the feasibility of interneuron-based therapy

Here, we also investigated how MGE transplant-derived interneurons affect outputs of local circuits, e.g., the spiking behavior of pyramidal cells. Interactions between synaptic excitation and inhibition are often presented in terms of a neuronal input-output function, also referred to as “gain control” (reviewed by Silver, 2010; Trevelyan et al., 2015). Two different patterns of inhibition underlie this gain control, and are provided by distinct interneuron subtypes that target peripheral dendrites (SST^+^) or proximal dendrites and soma (PV^+^), respectively. These are the same two interneuron subtypes generated from our MGE progenitor transplantations, and we demonstrated that they shift the frequency-current curve (I-F curve) to the right providing an inhibitory gain control to endogenous pyramidal neurons (Figs. 6*E*, 7*E*, 8*E* ). This phenomenon was not a gain-of-function effect because a similar but greater right shift was also found when we activated native interneurons (Fig. 6*E*). The difference in magnitude of the I-F shift is likely a reflection of a discrepancy in cell numbers. That is, the number of native interneurons in a given hippocampal slice is significantly larger than that of transplant-derived interneurons (Fig. 1*B*,*C*). In either case, I-F curve was right shifted in a parallel manner, suggesting that the introduced gain control primarily exerts its function in the subthreshold range and once the threshold is overcome, spiking behavior is solely determined by properties intrinsic to the host circuits. For this reason, while the inhibitory gain control offers protective effects during hyperexcitable network disease states such as during a seizure, it does not alter the dynamic range of spiking in pyramidal cells, which is important to encode functional information under normal conditions.

## Conclusion

Here, we presented data strongly arguing that MGE transplant-derived interneurons make functionally inhibitory connections with host neurons. Although interneuron complexity presents a potential problem for cell-based therapies, we showed that MGE-derived progenitors share the same developmental lineage, innervation preference, and subtype-specific synaptic characteristics as endogenous MGE-derived interneurons. This information not only suggests a working mechanism underlying the functional impact of MGE progenitor transplantation but also sheds light on their ability to form appropriate local connections in an established network.

## Synthesis

Reviewing Editor: Alfonso Represa, INSERM Decisions are customarily a result of the Reviewing Editor and the peer reviewers coming together and discussing their recommendations until a consensus is reached. When revisions are invited, a fact-based synthesis statement explaining their decision and outlining what is needed to prepare a revision will be listed below. The following reviewer(s) agreed to reveal their identity: Linda Overstreet-Wadiche

Dear Authors,

Your manuscript has been now revised and the changes/amendments evaluated positively. Some concerns still remain that you should take into account (see below). To not also that your rebuttal, notably the answer to the first reviewer' comment, removed the “incognito mode” but I understand that this was to justify that part of the questions evoked by the referee were analyzed before in your previous reports.

The authors have addressed some concerns raised by the reviewers to improve aspects of the manuscript. However, some issues remain.

1) The authors added additional immunohistochemical analysis to discern the marker expression profile of the non-PV and non-SST. However, they declined to fully characterize the intrinsic properties and anatomy of ChR2-expressing transplanted cells to determine their identity, and a new concern relates to the data added to Figure 1. The input resistance and spiking pattern are insufficient to identify the recorded cells as PV and SOMs. According to the cited reference (Fig 7, Table 3 of Tricoire et al., 2011) and other studies, PV+ fast spiking cells have lower input resistance than SOMs. The interneurons with average IR of 495 should not be classified as PV+. There is simply not enough information to rigorously classify these cells, so this data should be removed.

2) The authors have softened the conclusion about the degree of connectivity of implanted cells. It seems there is agreement that the current optogenetic approach does not assess the strength of unitary connectivity, so it is inappropriate to call the connections “strong” or discount the possibility that unitary connectivity is weaker from implanted cells (i.e.

Abstract, line 33; discussion, line 273). Such relative descriptions would require that the authors actually measure unitary connections. Although it is reasonable to assume that the smaller IPSCs and weaker inhibition results from the low number of transplanted neurons, that argument cannot be used to rule out differences in unitary connectivity.

The main novel finding here is that implanted cells appear to target appropriate postsynaptic domains of PCs, along with the additional evidence that transplanted interneurons selectively target principal cells. These results are convincing but somewhat narrow in terms of providing new insight into the function of interneuron transplants.

3) The functional measures of inhibition remain disconnected from the main finding that transplanted PV and SST interneurons maintain somatic and dendritic-projecting properties. The authors declined to discuss/address my prior technical concerns and presentation issues with these experiments. It also appears that functional inhibition is quite weak - equivalent to only 6% of existing inhibition (i.e. 21 pA shift in I-F compared to 343 pA shift shown in Figure 6), and the inhibition provided by SST cells is not convincing since the example does appear to be representative of the grouped data. Overall I find this part of the manuscript is weak in terms of confirming how interneuron transplantation provides subtype-specific inhibition (the last line of the abstract).
